# Social information changes stress hormone receptor expression in the songbird brain

**DOI:** 10.1016/j.yhbeh.2017.10.002

**Published:** 2018-01

**Authors:** Jamie M. Cornelius, Gillian Perreau, Valerie R. Bishop, Jesse S. Krause, Rachael Smith, Thomas P. Hahn, Simone L. Meddle

**Affiliations:** a Department of Biology, Eastern Michigan University, Ypsilanti, MI, USA; b The Roslin Institute, The Royal (Dick) School of Veterinary Studies, The University of Edinburgh, Easter Bush, UK; c Animal Behavior Graduate Group, Department of Neurobiology, Physiology and Behavior, University of California Davis, Davis, CA, USA

**Keywords:** Corticosterone, Mineralocorticoid receptor, Glucocorticoid receptor, Red crossbill, Food reduction

## Abstract

Social information is used by many vertebrate taxa to inform decision-making, including resource-mediated movements, yet the mechanisms whereby social information is integrated physiologically to affect such decisions remain unknown. Social information is known to influence the physiological response to food reduction in captive songbirds. Red crossbills (*Loxia curvirostra*) that were food reduced for several days showed significant elevations in circulating corticosterone (a “stress” hormone often responsive to food limitation) only if their neighbors were similarly food restricted. Physiological responses to glucocorticoid hormones are enacted through two receptors that may be expressed differentially in target tissues. Therefore, we investigated the influence of social information on the expression of the mineralocorticoid receptor (MR) and glucocorticoid receptor (GR) mRNA in captive red crossbill brains. Although the role of MR and GR in the response to social information may be highly complex, we specifically predicted social information from food-restricted individuals would reduce MR and GR expression in two brain regions known to regulate hypothalamic-pituitary-adrenal (HPA) activity - given that reduced receptor expression may lessen the efficacy of negative feedback and release inhibitory tone on the HPA. Our results support these predictions - offering one potential mechanism whereby social cues could increase or sustain HPA-activity during stress. The data further suggest different mechanisms by which metabolic stress versus social information influence HPA activity and behavioral outcomes.

## Highlights

• Animals use social information to inform decisions.
• Food-restricted neighbors induce enhanced corticosterone release during food restriction.
• Mechanisms for how social information affects changes in behavior are unknown.
• We propose changes in HPA-regulation via corticosterone receptors as one mechanism.
• Reduced receptor expression in key brain regions may sensitize HPA axis and underlie social effect.

## 1 Introduction

Individuals must make behavioral and physiological adjustments as environmental conditions change if they are to survive and reproduce successfully. Animals use a wide variety of information sources to assess environmental conditions – the most obvious being through direct experience, such as assessment of food availability as an individual animal attempts to assimilate energy from the environment (i.e., optimal foraging) (Charnov, 1976, Pyke et al., 1977). However, animals also use indirect assessments to inform decision-making. Social information, or information obtained through observation of and/or communication with other individuals is an important indirect information source used by animals to assess conditions and make decisions (Valone, 1989, Valone and Templeton, 2002). For example, social information can change the duration individuals forage on a patch if other individuals are present to provide information (Smith et al., 1999, Templeton and Giraldeau, 1995) and can influence large-scale, facultative migratory responses to resources as well (Chan, 1994). The physiological mechanisms for how such social information might influence behavioral outcomes, however, remain poorly understood - though some evidence suggests that the endocrine system may be involved (Cornelius et al., 2010).

The endocrine system acts as a broad-scale messenger between neural processing centers and the body and, thus, provides a tool for measuring physiological responses to different environmental and endogenous conditions. Corticosterone, for example, is a steroid hormone implicated in metabolic processes and survival behaviors during food shortage (Astheimer et al., 1992, Cornelius et al., 2010, Fokidis et al., 2012, Fokidis et al., 2011, Kitaysky et al., 1999, Kitaysky et al., 2010, Krause et al., 2017, Lynn et al., 2003, Lynn et al., 2010). The amount of corticosterone that is released in response to a physical or metabolic stressor is regulated by the hypothalamic-pituitary-adrenal (HPA) axis, which is itself under neural control from multiple processing centers of the brain (de Kloet, 2014, Joels et al., 2008, Sapolsky et al., 2000). The elevation in corticosterone levels during food restriction is likely due to detection of metabolic imbalance (e.g., plasma fatty acids in birds) and subsequent stimulation of the HPA via adrenergic or cholinergic inputs (Calogero, 1995, Landys et al., 2004a, Landys et al., 2004b, Tsagarakis et al., 1988). Plasma corticosterone then coordinates broad scale cellular responses that influence metabolism and behavior. For example, increased plasma corticosterone has been linked to fatty acid metabolism in birds (Landys et al., 2004a, Landys et al., 2004b) and has been correlated with enhanced activity in captive birds when food is absent or unpredictable (Astheimer et al., 1992, Cornelius et al., 2010, Krause et al., 2017), altitudinal migration in wild birds to escape bad weather when food becomes unavailable (e.g., covered in snow) (Breuner and Hahn, 2003) or when conditions are extreme (Krause et al., 2016). For corticosterone to achieve these effects it must bind with receptors at the target tissues.

Hormone receptors detect the presence of the hormone message and instruct the receiving cell to respond in a target-specific manner. Increasing the system's complexity, these receptors are expressed in brain regions influencing HPA activity such that the binding of corticosterone can inhibit HPA-activity (i.e., negative feedback) regardless of the continued presence of a stressor or stimulus (de Kloet, 2014, Joels et al., 2008, Sapolsky et al., 2000). Ultimately the amount of corticosterone circulating in the blood stream is controlled by the neural networks that stimulate corticosterone release and those that regulate negative feedback – both of which can be influenced by the two corticosterone receptors: the high affinity mineralocorticoid receptor (MR) and the low affinity glucocorticoid receptor (GR) (Breuner and Orchinik, 2009, Lattin et al., 2012).

MR is predominately expressed in the hippocampus in birds and mammals (Baugh et al., 2017, de Kloet, 2014, Dickens et al., 2009, Hodgson et al., 2007, Joels et al., 2008, Krause et al., 2015, Senft et al., 2016, Zimmer and Spencer, 2014). In the rodent brain MR are activated by basal concentrations of corticosterone and are thought to regulate basal levels of corticosterone with an overall inhibitory tone (Reddy et al., 2009, Zilliacus et al., 1995). Studies in rodents further indicate that MR can be targeted for insertion in the cellular membrane and this form is thought to act similarly to the low affinity GR receptor to help facilitate the stress response by promoting rapid behavioral responses to elevated plasma corticosterone (Joels et al., 2008). The low-affinity GR is a nuclear receptor with transcriptional activity that is predominately expressed in the paraventricular nucleus, amygdala, cerebral cortex and the hippocampus in rodents (de Kloet, 2014, Joels et al., 2008, Sapolsky et al., 2000). Similar patterns of GR exist in birds except for expression in the hippocampus, which seems more variable across species (Baugh et al., 2017, Hodgson et al., 2007, Krause et al., 2015, Senft et al., 2016, Zimmer and Spencer, 2014). GR becomes activated in mammals when corticosterone levels rise during the circadian peak, during a stress response or when metabolic demands rise (Reddy et al., 2009, Reul and de Kloet, 1985, Zilliacus et al., 1995). Changes in MR and GR expression in the avian brain have been correlated with seasonal changes in hormone profiles, selection for high stress responsiveness, and under chronic stress (Dickens et al., 2009, Hodgson et al., 2007, Krause et al., 2015). More importantly, food restriction in rodents has been shown to reduce receptor expression in the brain leading to reduced negative feedback and elevated levels of corticosterone (Lee et al., 2000). The interaction between circulating levels of corticosterone, MR and GR expression in the brain and social information to regulate physiology and decision-making processes, however, remain completely unexplored in any taxa.

Cornelius et al. (2010) previously demonstrated that social information can alter corticosterone secretion in a bird that is highly adapted to coping with unpredictable food resources. Red crossbills (*Loxia curvirostra*) that were provided with 75% of their average daily food intake had larger increases in plasma corticosterone if their neighbors were similarly food reduced, compared to those whose neighbors were given food ad libitum. Here we investigate a potential role for corticosteroid receptors in the response to social information and food cues. We call special attention to brain regions that regulate the sensitivity of the HPA axis: the hippocampus and paraventricular nucleus (PVN). We predict that social information from food-deprived individuals reduces inhibition of the HPA-axis, thereby allowing the system to respond more aggressively to food stress. Further, if alterations in these receptor populations are involved in processing of the food and social cues in this context, we predict that social information will enable enhanced HPA-activity during food limitation by reducing expression of MR and GR in these regions.

## 2 Methods

### 2.1 Birds and experimental outline

**Fig. 1:**
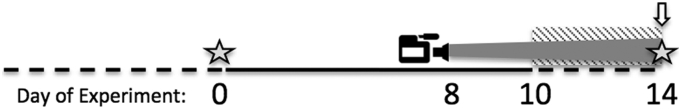
Experimental timeline. Daily food intake (dashed line) was measured for one week prior to the start of the experiment. Body condition and CORT (stars) were collected on days 0 and 14. Activity (camera beam) was filmed daily from days 8 to 14. Food was reduced (hashed box) in the food treatment group beginning on day 10, which marks the change from the pre-treatment to treatment phase of the experiment. Brains were collected on day 14 (open arrow).

This experiment replicates the methods described in Cornelius et al. (2010), with a few exceptions as described below. Red crossbills occur as a suite of eco-types that differ in vocal call structure and body/bill size. Type 3 is the smallest eco-type specializing on small, soft-coned conifers and is known to exhibit mass migratory movements related to food availability. Briefly, 32 adult male type 3 red crossbills were captured on the Olympic Peninsula in Washington State (47° 27′ 25.92″ N, − 123° 43′ 54.48″ W), USA, in July 2008 and transported to facilities at University of California-Davis. Males were used exclusively to avoid potentially confounding effects of mixed sex pairs and to preserve sample sizes within treatment groups. Based on prior results (Cornelius et al., 2010) we do not predict males to have a qualitatively distinct corticosterone response relative to females in this experimental design, though females would need to be tested to determine if there are sex-specific differences in receptor mRNA expression. The experiment was performed in February and March 2008 and birds were housed on naturally changing photoperiod. This is a time of year that natural declines in conifer seed supplies may require these birds to move (Cornelius and Hahn, 2012, Hahn, 1998). All birds were kept in individual cages for two months prior to any manipulations on an ad libitum diet of Roudybush pellet food and a daily allotment of 2 pine nuts per bird. Morphological and hormone data were collected from each individual in a repeated measures design with pre-treatment (Day 0) and treatment (Day 14) sampling points (Fig. 1). All experiments were approved by University of California Davis Institutional Animal Care and Use Committee under protocol 05-12095.

### 2.2 Experimental treatments

Individuals were housed on shelves in acoustic isolation chambers (IAC 250 “Mini” Sound Shelters, 61 cm wide by 86 cm deep by 168 cm high inside dimensions; Industrial Acoustics Company, Bronx, New York) with visual and acoustic access to a single neighboring bird. Four social and food treatment groups were created by randomly assigning individuals to the following housing configurations: ad libitum subject with an ad libitum neighbor A(a), ad libitum subject with a food reduced neighbor A(f), food reduced subject with an ad libitum neighbor F(a), and food reduced subject with a food reduced neighbor F(f).

“Food Reduced” individuals had their food restricted to 75% of their average daily intake of food pellets and they received no pine nuts during the treatment period (Days 10–14; Fig. 1). Food reduction caused an average loss in body mass of 5 g (i.e., 18% of average body mass; range 3 to 8 g) in experimental birds, with the exception of one bird that did not lose body mass during food reduction and was therefore a statistical outlier (> 2 SD from the mean change in body mass) for food-reduced birds. It is highly probable that the average daily intake was over-estimated for this individual (e.g., food lost from covered food cup without the investigator detecting the loss) and a subsequent 75% reduction did not metabolically challenge this bird. This is further supported by the fact that this bird did not consume all of its allotted food during the first two days of the food reduction. We removed this bird from further statistical analyses thus all analyses (except corticosterone, see below) contained a sample size of 8 birds each in A(a), A(f) and F(a) and 7 birds in the F(f) group.

### 2.3 Activity data

Activity data were collected using a Panasonic mini-DV recorder. Activity levels were scored on the video by dividing the cage into quadrants and recording the number of times the bird entered a new quadrant during an 80-minute filming session. Final activity levels reflect an average of three sessions filmed across 3 days for each of the pre-treatment and treatment sampling periods (Fig. 1). Time of filming for each individual was held constant across all sampling periods and all video was collected within 4 h of lights-on. Activity was scored blind with respect to subject identification and sampling period. The proportional change in activity between the pre and post treatment phases was used in all analyses to control for individual differences in general activity levels.

### 2.4 Body condition and blood/tissue samples

Body mass was measured to the nearest 0.5 g using a Pesola spring scale and furcular and abdominal fat were ranked on a scale of 0 (no fat) to 5 (bulging; after Helms and Drury, 1960). Baseline blood samples were collected as a measure of recent HPA-activity in the different treatment groups. Eighty microliters of blood were collected into heparinized microhematocrit capillary tubes following puncture of the alar vein with a sterile 26-gauge needle. These samples were collected within 3 min of opening the door of the isolation chamber to minimize the influence of capture and handling stress on baseline corticosterone levels (Wingfield et al., 1982). Three samples out of 64 were not collected within 3 min and were excluded from the analysis (one A(f), one F(a) and one F(f)). Time-of-day of sampling was standardized for each individual across the sampling periods, and all individuals were sampled between 07:30 and 10:30. Plasma samples were stored on ice until centrifugation, at which point the plasma was separated from the cellular fraction. Plasma samples were kept at − 80 °C until the corticosterone assay was performed.

Following collection of the Day 14 blood sample, all birds were immediately anesthetized with isoflurane, decapitated, and brains extracted and frozen on dry ice. Average time from initial capture to anesthesia was 16 min (range 5–27 min) and 4.4 min (range 3.7 to 5.8 min) from anesthesia to freezing of tissue. It is unknown if receptor mRNA expression can change in response to this range of handling time, but handling order was balanced across treatment groups to preclude a sampling bias of handling time (average time from handling to anesthesia in our four groups was 15, 14.5, 15.4, 17.2 min; ANOVA P = 0.88). Whole brains were kept at − 80 °C and later shipped frozen on dry ice to the Roslin Institute, University of Edinburgh where they were stored at − 70 °C until they were processed for GR and MR mRNA quantification by in situ hybridisation.

### 2.5 Corticosterone assay

Plasma corticosterone concentrations were determined using radioimmunoassay as described previously (Wingfield et al., 1992). Tritiated corticosterone was purchased from Perkin Elmer (NET399250UC), antibody from MP Biomedical (07-120016, Lot 3R3-PB-20E) and scintillation fluid from Perkin Elmer (Ultima gold 6013329). Samples were counted using a Beckman 6500 liquid scintillation counter. Samples were run in two consecutive assays with mean recoveries of 90% and 91% and a mean detection limit of 0.9 ng/mL and 0.8 ng/mL, respectively. Inter-assay variation was 6.5% and intra-assay variation was 5.59% (range 0.02–33%, median 2.45%). Five of 64 samples exceeded a maximum cut-off of 20% CV, but these samples were all very low in concentration (< 2 ng/mL) and their inclusion did not alter the statistical outcomes for corticosterone analyses and they were therefore retained to preserve sample sizes for other variables in the model. The bound to free ratio was 0.28 for assay 1 and 0.30 for assay 2.

### 2.6 In situ hybridisation histochemistry for MR and GR mRNA

Whole brains were sectioned coronally at 15 μm on a cryostat and thaw mounted onto polysine, RNAase free, pre-treated glass microscope slides. Marker slides were created by collecting every twentieth section and then stained using a cresyl violet. Sections were stored at − 70 °C with silica pellets until the in situ hybridisation was performed. Slides for hybridisation work were selected after examination of marker slides in conjunction with the canary stereotaxic atlas (Stokes et al., 1974) to locate regions of interest. The MR and GR hybridisation procedures have been described in detail previously (Dickens et al., 2009, Hodgson et al., 2007, Krause et al., 2015). Briefly, 500-bp fragments of the zebra finch GR (Genbank: DQ864494) or MR (Genbank: DQ539433) were subcloned into PGEM-7. GR sense and antisense riboprobes were generated by in vitro transcription, in the presence of 35S-UTP, with SP6- and T7-RNA polymerase after plasmid linearisation with *Eco*RI or *Hin*dIII, respectively. MR sense and antisense riboprobes were generated by in vitro transcription, in the presence of 35S-UTP, with T7- and SP6-RNA polymerase after plasmid linearization with HindIII or *Apa*I, respectively. The clones were generously provided by Drs M. Gahr and R. Metzdorf (Department of Behavioural Neurobiology, Max-Planck-Institute for Ornithology, Seewiesen, Germany). Both GR and MR are highly conserved genes, with identities between zebra finch (passerine) and chicken (galliform) of 88% and 90%, respectively (Dickens et al., 2009).

Slides were dipped in autoradiography emulsion to visualise the hybridized cells and exposed for 6 weeks. Autoradiographs were developed, counterstained with haematoxylin and eosin and cover-slipped with DPX mountant (Sigma, St Louis, MO, USA). Hybridisation of sections with GR or MR sense riboprobes, or pre-treatment with RNase-A prior to hybridisation with the GR or MR antisense riboprobes, produced no detectable hybridization signal.

### 2.7 Quantification of MR/GR mRNA expression

Slides were coded so the identity of each bird was unknown during analyses. Silver grain density was quantified in the autoradiograph; it appears black under bright field microscopy. Anatomical structures were determined in combination with the canary stereotaxic atlas (Stokes et al., 1974) and marker slides to locate brains regions of interest (ROI) containing the PVN, hippocampus and nucleus septalis medialis (LS). GR mRNA was quantified in the PVN and MR mRNA was quantified in the hippocampus (e.g., Dickens et al., 2009, Krause et al., 2015, Krause et al., 2015, Hodgson et al., 2007, Baugh et al., 2017, Senft et al., 2016). Photographs of the region of interest (ROI) in both the left and right side of the brain were taken on a Nikon E600 (Nikon Co., Ltd) microscope, using Scion Visicapture. Images were captured at a four-fold magnification, with all settings kept constant throughout the capturing process. The mRNA in the ROI in silver grain density/mm^2^ was measured with IMAGEJ (NIH, Bethesda, MD, USA). A background measurement was also taken from each image and subsequently subtracted from the ROI measurement to eliminate the effect of different background levels on the accuracy of the final value (e.g., Senft et al., 2016).

### 2.8 Statistical analyses

Effects of food reduction, social treatment, date and their interactions on body condition (i.e., mass and fat) and activity were analyzed in a general linear mixed model with repeated measures of our fixed effects and individual included as a random effect (Table 1). Post-hoc ANOVA and regression were used to compare significant predictor variables among the four treatment groups. All analyses were performed in JMP® (Version *13.0*, SAS Institute Inc., Cary, NC, 1989–2007). Residuals for the change in baseline corticosterone (ng/mL) and absolute measures of GR and MR mRNA expression data were not distributed normally (P < 0.05 Shapiro-Wilks test) and could not be normalized sufficiently with transformation to warrant parametric statistics. Differences between treatment groups were therefore analyzed using non-parametric Kruskal-Wallis (corticosterone) or Wilcoxon (MR/GR) tests. Hierarchical predictions were further tested using the Jonckheere test for ordered alternatives (Cornelius et al., 2010).

**Table 1:** GLMM results for activity, mass and fat.

	Activity			Mass			Fat		
Fixed effects	DF	F	P	DF	F	P	DF	F	P
Date	1	6.74	**0**.**01**	1	155.4	<** 0**.**0001**	1	42.8	<** 0**.**0001**
Food treatment	1	4.69	**0**.**03**	1	0.06	0.81	1	2.75	0.11
Food treatment ∗ date	1	4.69	**0**.**03**	1	28.4	<** 0**.**0001**	1	16.7	**0**.**0003**
Social treatment	1	1.68	0.20	1	3.7	0.07	1	2.75	0.05
Social treatment ∗ date	1	1.68	0.20	1	0.16	0.69	1	0.06	0.79
Food treatment ∗ social treatment	1	1.57	0.21	1	0.04	0.84	1	0.00	0.95
Food treatment ∗ social treatment ∗ date	1	1.57	0.21	1	0.08	0.77	1	0.19	0.66
Individual (random)			1.00			0.0004			0.0009

Bold numbers indicate significance at P values (< 0.05).

## 3 Results

### 3.1 Condition measures and behavior

**Fig. 2:**
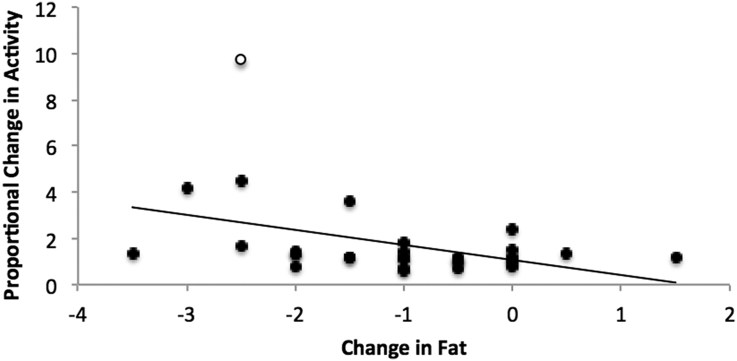
Relationship between body mass and activity during food treatment. The change in size-corrected body mass during food treatment negatively predicted the change in activity (F_31_ = 8.5, P = 0.007, r^2^ = 0.23). The outlier (open circle) did not influence the statistical significance of the pattern.

Food treatment significantly impacted activity, fat and mass between days 0 and 14 (Table 1). Captive birds reduced to 75% of their daily food intake experienced a decline in mass (t_31_ = 16.1, P < 0.0001) and fat deposits (t_31_ = 18.0, P = 0.0002) and increased activity (t_31_ = 4.89, P = 0.03). Regression analysis revealed that the proportional change in activity was negatively related to the change in mass (F_30_ = 8.01, P = 0.008, r^2^ = 0.21) and fat (Fig. 2; F_30_ = 6.1, P = 0.02, r^2^ = 0.17), such that individuals experiencing a larger loss of body condition were also more active, though the relationship becomes non-significant for mass when the outlier is excluded (F_29_ = 2.4, P = 0.12, r^2^ = 0.08). Birds in the social treatment with food reduced neighbors began the experiment with slightly lower fat deposits (Table 1; average with control neighbors 6.5, average with reduced neighbor 5.4; t_31_ = − 2.3, P = 0.02), but there were no interactive effects of social treatment with sample date or food treatment on body condition measures or activity levels.

### 3.2 Corticosterone

**Fig. 3:**
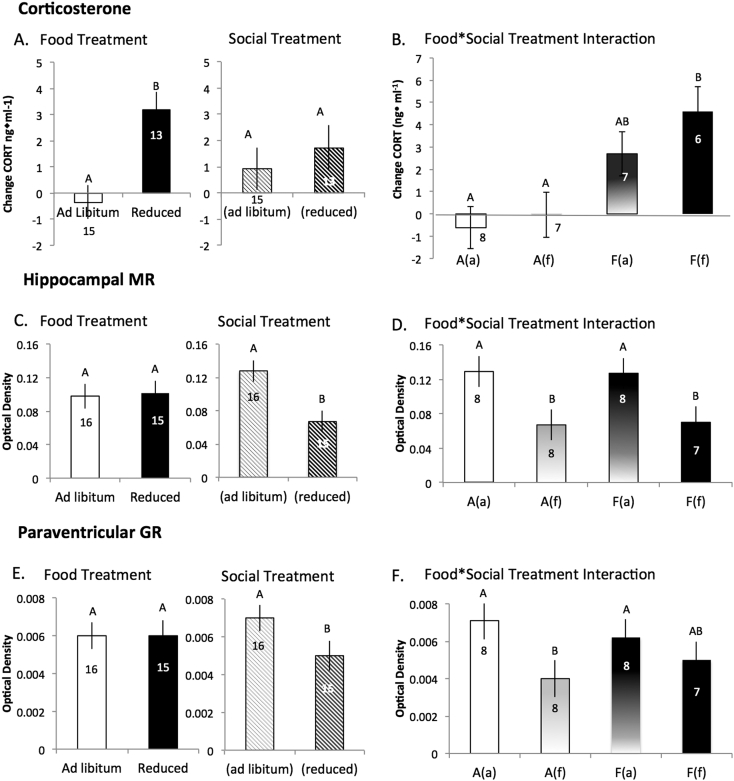
Effect of food and social treatments on plasma corticosterone concentrations and glucocorticoid and mineralocorticoid receptor mRNA expression in captive red crossbills. Food reduction caused an increase in plasma corticosterone whereas social information had no effect (Panel A). There was a significant interaction between food and social treatment such that corticosterone increased hierarchically across groups (Panel B) and was significantly higher in food reduced birds if the social informant was similarly food reduced (i.e., F(f) group), but not significantly so if the social informant was fed ad libitum (i.e., F(a) group). Optical densities for hippocampal MR mRNA (Panels C, D) and paraventricular GR mRNA (Panels E, F) are summarized by food treatment only or social treatment only (Panels C, E). Food treatment had no influence on expression in the brain regions measured, whereas social treatment significantly influenced mRNA expression: birds with food-reduced neighbors had significantly lower MR mRNA expression in the hippocampus (HP) and GR mRNA expression in the paraventricular nucleus (PVN). There was no hierarchical effect or interaction between food and social treatment on either MR or GR expression (Panels D, F). Bars represent group averages with ± sem. Sample sizes given and letter groups denote significantly different groups by Wilcoxon or Kruskal Wallis rank sum test.

Food reduction caused significant elevations in baseline plasma corticosterone (Fig. 3A; χ^2^ = 13.2; P = 0.0003). In agreement with the Cornelius et al. (2010) study, the change in baseline corticosterone during the 4 day treatment period was also significantly affected by the interaction between food and social treatment (Fig. 3B; Kruskal-Wallis χ^2^ = 13.5, P = 0.004) and the Jonckheere test for ordered alternatives found the data to support the predicted hierarchical response: A(a) < A(f) < F(a) < F(f) (Jonckheere P = 0.0026).

### 3.3 Distribution of MR and GR mRNA expression

**Fig. 4:**
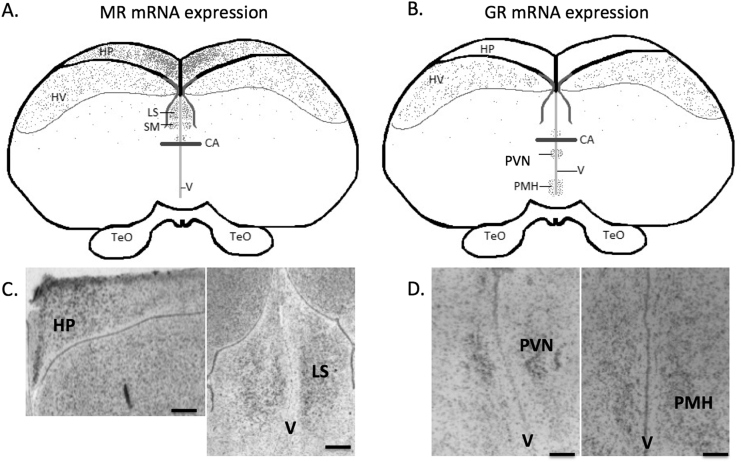
Distribution maps (A & B) and representative photomicrographs of autoradiographs in bright field (C & D) of glucocorticoid receptor (GR) A & C and mineralocorticoid receptor (MR) B & D mRNA expression in the red crossbill brain. Hippocampus (HP), hyperstriatum ventrale (HV), lateral septum (LS), nucleus septalis medialis (NSM), nucleus medialis hypothalamic posterioris (PMH) and paraventricular nucleus (PVN). Commissura anterior (CA), third ventricle (V) and tectum optium (TeO) are provided for reference. Scale bars = 50 μm (C & D).

MR mRNA was expressed in multiple regions of the telencephalon, including the hippocampus (HP), hyperstriatum ventrale (HV), lateral septum (LS) and nucleus septalis medialis (NSM). GR mRNA was found in the paraventricular nucleus (PVN) and nucleus medialis hypothalamic posterioris (PMH) in the diencephalon (Fig. 4).

### 3.4 Treatment effects on MR and GR mRNA expression

MR and GR expression were not affected by time between capture and euthanasia, nor by time between capture and freezing of tissue (results not shown). Food treatment had no detectable effect on expression of either MR or GR mRNA in any of the brain regions measured (Fig. 3C, E; Wilcoxon P > 0.05). Social treatment, however, influenced expression of GR mRNA in the PVN, and of MR mRNA in the hippocampus (Fig. 3C, E; Wilcoxon P < 0.05). Specifically, birds with food-reduced neighbors (i.e., A(f) and F(f)) had less dense MR expression in the hippocampus and GR expression in the PVN relative to birds with ad-libitum fed neighbors (i.e., A(a) and F(a)) (Fig. 3C, E). There was no interactive effect of food and social treatment (i.e., hierarchical response) on either MR or GR mRNA expression (Fig. 3D, F; Jonckheere P = 0.99). There was no significant correlation between baseline plasma corticosterone levels and GR mRNA expression in the PVN or MR expression in the hippocampus (F = 0.47, P = 0.49, r^2^ = 0.02 and F = 2.12, P = 0.15, r^2^ = 0.08, respectively), nor was there a relationship between GR and MR expression and activity (F = 0.16, P = 0.70, r^2^ = 0.005 and F = 0.19, P = 0.67, r^2^ = 0.007, respectively).

## 4 Discussion

Social information from food-manipulated neighbors reduced mRNA expression of MR in the hippocampus and of GR in the PVN. This is the first time to our knowledge that social cues have been found to alter corticosteroid receptor mRNA expression in a vertebrate animal. Food restriction, on the other hand, did not influence GR or MR mRNA expression in the brain regions examined. These results stand in contrast to the more complex response of plasma corticosterone to food restriction and social cues, whereby food restriction induced a stronger corticosterone response if the neighbor was similarly food reduced but control birds showed no change in corticosterone regardless of social treatment. The fact that social information caused reductions in receptor expression in these regions that are often associated with negative feedback sensitivity suggests the possibility that reduced inhibitory tone through MR in hippocampus and reduced negative feedback through GR in the PVN may be responsible for the interactive effect between food, social treatments and corticosterone levels in our study. Information from an informant with limited food resources may be important for priming neural networks either by changing homeostatic setpoints for hormonal signaling or through changes in neural pathways for decision-making processes (especially in the hippocampus). The absence of elevated corticosterone levels in the ad libitum bird paired with a food restricted neighbor suggests a system in which metabolic demands have remained unchanged, resulting in no change in HPA activity despite the change in mRNA receptor expression. In contrast, if food availability were to change rapidly for that individual the system would already be primed for rapid changes in corticosterone production.

This study corroborated earlier findings that social information influences HPA activity during a food challenge in red crossbills (Cornelius et al., 2010). Such a response may enhance the accuracy of resource assessment for a given individual or aid group decision making in cohesive societies by bringing group members into more similar physiological states (Cornelius et al., 2010). Corticosterone is thought to enhance survival during environmental stress by increasing foraging behavior or exploration and assessment of the local environment and by suppressing reproductive investments (for review see (Blas, 2015). In the extreme it may underlie decisions to leave a current known, but unsatisfactory foraging region for an unknown but potentially superior alternative. This decision is inherently risky, thus there is probably strong selective pressure on mechanisms that reduce mistakes or enhance environmental assessment. The finding that social information, independent of direct foraging experience, may alter HPA-sensitivity through MR and GR mRNA expression provides a mechanism by which birds like red crossbills may incorporate social information into resource-mediated decisions. These results also highlight the idea that reduced negative feedback within the HPA can be adaptive in certain acute stress contexts such as declining food, despite the potential negative implications for health in chronic stress and disease contexts (Pariante and Lightman, 2008, Wirtz et al., 2007).

We did not detect a correlative relationship between the change in plasma corticosterone during the treatment phase of the study and MR or GR mRNA expression, in agreement with one other study in birds (J. Krause et al., 2015). This stands in contrast with studies in mammals, which have been conducted largely from a mechanistic standpoint using laboratory models that have evaluated the influence of ablation or competitive inhibition of receptors on hypothalamic output (Bouillé and Baylé, 1976, Jacobson and Sapolsky, 1991, Ratka et al., 1989, Van Haarst et al., 1996). An emerging field of avian studies has used a more ecological approach, investigating changes in MR or GR mRNA expression in wild birds with regard to particular behavioral, ecological or physiological contexts. Despite some known similarities between structure and function of the involved brain regions in birds and mammals, these field studies of birds are not yet easily interpreted in terms of known patterns in mammals. For example, many arctic breeding passerines transitioning from a pre-parental (i.e., territory forming and mate defending) phase of life into the parental phase typically show a strong reduction in HPA sensitivity to stress, which is thought to facilitate the behavioral transition to parental behavior (Holberton et al., 2003, Krause et al., 2015, Meddle et al., 2003, Wingfield et al., 1995). Based on mammalian literature, this pattern would predict an increase in MR and GR expression in the hippocampus and PVN, respectively, given that the HPA axis apparently becomes less sensitive to stressors during the parental phase (i.e., stronger inhibitory tone). However, MR expression in the hippocampus decreased and GR expression remained static in the arctic songbirds (J. Krause et al., 2015). Similarly, European starlings that typically show an attenuated stress response during chronic stress show decreased GR and MR mRNA expression in PVN and hippocampus, respectively – though in this case chronic stress may have upset normal HPA functioning (Dickens et al., 2009). There is some evidence suggesting that the ratio between MR and GR is important to HPA activity, which may also account for the differences observed between mammalian and avian studies (de Kloet, 2014, Harris et al., 2013), but more research is clearly needed to link MR and GR expression to HPA activity in birds. Finally, changes in mRNA expression may or may not directly correlate with changes in protein expression (Medina et al., 2013). There are many intermediary steps that can alter the relationship between translation and protein expression. Adjustments within this complex pathway may further explain the lack of a relationship between plasma corticosterone and mRNA expression measured in this study.

No published studies on birds have investigated the effects of fasting on MR or GR mRNA expression, but receptor antagonist studies suggest that GR plays a role in hyperphagia and lipid mobilization during energy challenges such as fasting or migration (Landys et al., 2004b). Food reduction did not change the expression of MR or GR mRNA in the brain regions measured in this study, though it had a strong effect on HPA output and behavior as measured by increased plasma corticosterone and increased activity. The effects of food treatment and social information on HPA activity are apparently occurring through different mechanisms whereby food reduction triggers HPA activation and social information alters HPA sensitivity. While both mechanisms may influence behavior, food reduction was the only cue stimulating increased activity in this study – similar to the pattern seen in plasma corticosterone levels. As in the Cornelius et al. (2010) study, behavior did not respond in an interactive way with social treatment. This may reflect small sample sizes being unable to detect real differences between food and social treatments, or simply be the consequence of the complexity of behavioral decisions. Activity increased in proportion to the amount of mass and fat lost during food reduction, suggesting a mechanism involving body condition as well as metabolic state. There was similarly no direct relationship between MR or GR mRNA expression and behavior. This was not particularly surprising given the complexity of behavioral decisions and further suggests that MR and GR influence behavior indirectly through impacts on HPA sensitivity.

## 5 Conclusion

Social information changed receptor mRNA production in the brains of male red crossbills, demonstrating that the observation of or communication with other individuals can impact brain physiology. The generality of this result to female red crossbills or other species has not yet been determined, but social information is used by a wide array of species in a wide array of contexts (Danchin et al., 2004) and the HPA-axis is highly conserved among vertebrates (Chang and Hsu, 2004, Wingfield and Romero, 2001). Stress hormone receptor expression may therefore be one mechanism whereby neighbors – or social information – tune an individual's response to changing environmental conditions.
